# Growth conditions that increase or decrease lifespan in *Saccharomyces cerevisiae* lead to corresponding decreases or increases in rates of interstitial deletions and non-reciprocal translocations

**DOI:** 10.1186/s12863-016-0447-5

**Published:** 2016-10-21

**Authors:** Patrick H. Maxwell

**Affiliations:** Department of Biological Sciences, Rensselaer Polytechnic Institute, CBIS Room 2123, 110 8th Street, Troy, 12180 NY USA

**Keywords:** Aging, Chromosomal rearrangement, Chronological lifespan, Mutation, Recombination, Retrotransposition, *Saccharomyces cerevisiae*

## Abstract

**Background:**

Accumulation of DNA damage, mutations, and chromosomal abnormalities is associated with aging in many organisms. How directly various forms of genomic instability contribute to lifespan in different aging contexts is still under active investigation. Testing whether treatments that alter lifespan change mutation rates early during lifespan could provide support for genomic instability being at least partly responsible for changes in the rates of aging.

**Results:**

Rates of mutations, direct repeat recombination, or retrotransposition were measured in young cell populations from two strain backgrounds of *Saccharomyces cerevisiae* exposed to several growth conditions that shortened or extended yeast chronological lifespan. In most cases, rates of genomic instability did not consistently increase in young cells exposed to lifespan-shortening conditions or decrease in young cells exposed to lifespan-extending conditions. The mutation rate for a copy of the *CAN1* gene integrated onto the right arm of chromosome VIII did show expected increases or decreases in young cells in the lifespan-altering growth conditions. These mutations were determined to frequently result from non-allelic recombination events, including non-reciprocal translocations, and were more strongly stimulated by using hydroxyurea to induce DNA replication stress than by the general DNA-damaging agent methyl methanesulfonate.

**Conclusions:**

The results are not consistent with changes in mutation rates in general mediating the influence of alternative growth conditions on yeast lifespan. The strong correlation between non-allelic recombination events and the effects of the alternative growth conditions on lifespan indicates that genomic instability due to changes in recombination rates may directly contribute to the rate of aging or that lifespan-altering treatments may consistently increase or decrease DNA replication stress. These results further support the connection between DNA replication stress and aging observed in multiple organisms. Chromosomal abnormalities that likely arise from recombination events are more prevalent in multiple human tissues with increasing age, and further work in yeast could help to define mechanisms responsible for this observation and the impact of chromosomal abnormalities on aging.

**Electronic supplementary material:**

The online version of this article (doi:10.1186/s12863-016-0447-5) contains supplementary material, which is available to authorized users.

## Background

Frequent references are made to the contribution of DNA damage and mutations to the aging process, but a number of questions remain regarding the precise relationship between genomic instability and aging. Evidence consistent with a role for genomic instability in aging includes increases in specific forms of DNA damage, small-scale mutations, and chromosomal rearrangements with age in *Saccharomyces cerevisiae, Drosophila melanogaster*, rodents, and in cells from people of different ages [1–6]. The magnitude of the increase in mutations or whether mutations increase at all with age varies by tissue in mice [2, 3, 7, 8]. Some DNA repair activities have been reported to be less efficient with age, such as base excision repair, homologous recombination, and non-homologous end joining in mammalian cells and tissues [6, 9–14]. Changes in the activities of DNA damage signaling pathways have also been observed in aging mammalian cells [15, 16]. Human progerias associated with symptoms of premature aging are typically caused by mutations in single genes that compromise DNA repair and replication, and mice with mutations in certain DNA repair genes have shortened lifespans [17–20]. How shortened lifespan due to inherited deficiencies in DNA repair activities relates to normal aging has been questioned [20], but increased production of the progerin protein responsible for Hutchinson-Gilford Progeria Syndrome occurs in response to telomere shortening during aging of normal human fibroblasts [21].

The influence of age on frequencies of chromosomal abnormalities in human tissue samples has been analyzed using single nucleotide polymorphism (SNP) arrays in multiple studies. Chromosomal loss of heterozygosity events in human colon crypts are more frequent in older individuals [22]. A few studies identified that mosaic chromosomal abnormalities in human blood and buccal samples are detected at frequencies of less than 0.5 % in individuals under 50 years of age but at frequencies of ~2-3 % in individuals 75 years of age or older [23–25]. A high burden of single nucleotide variants has been observed in human neurons by sequencing genomes of individual cells (up to ~1,500 per genome), but the influence of age on accumulation of these variants remains to be determined [26].

While these and other observations are consistent with genomic instability promoting aging, one challenge is showing that improved DNA repair and decreased genomic instability directly extend lifespan. Part of the reason this is challenging is that many DNA repair activities involve numerous proteins, so overexpressing a single DNA repair gene may not improve repair [6]. Extension of lifespan in mice through dietary restriction ameliorates the age-related decrease in base excision repair [9], and extension of lifespan in yeast by inhibition of growth signaling reduces mutation accumulation during aging [27], but it is unclear whether these changes directly contribute to lifespan extension in these models. Mice with extended or shortened telomeres have corresponding increases or decreases in lifespan [28–30], and activation of telomerase in mice can delay normal aging [31, 32], but these studies do not address the role of other aspects of genomic instability in aging.

If genomic instability directly promotes aging, then levels of genomic instability early during lifespan should influence later accumulation of characteristics of aging and at least partly determine lifespan. Manipulations that alter lifespan would be expected to change the rate of accumulating mutations in young cells or organisms, causing them to age at different rates. Dietary restriction has been shown to increase base excision repair in young mice [9], and further examination of genomic instability early during lifespan in response to manipulations that alter lifespan could help address the role of DNA damage and mutations during aging.

Genomic instability during aging has been examined using two aging models for *Saccharomyces cerevisiae*. Chronological aging occurs as non-dividing cells in stationary phase lose function/viability [33], and is associated with increased small-scale mutations, chromosomal rearrangements, and mobility of retrotransposons [34, 35]. Replicative aging occurs as yeast mother cells undergo subsequent rounds of cell division [33], and is associated with increased loss of heterozygosity related to instability of the rDNA region as well as increased mobility of retrotransposons [36–39]. Mutations that inhibit double-strand break repair or that compromise DNA replication shorten yeast replicative lifespan [40, 41]. In contrast, large-scale screens for mutations that influence yeast chronological lifespan (CLS) have not identified DNA repair genes as major regulators of lifespan [42, 43]. A variety of changes to media and growth conditions have been demonstrated to alter lifespan in yeast, including increasing or decreasing the concentration of glucose, use of non-fermentable carbon sources, high medium osmolarity, and changing the growth temperature [44–48]. The current study addresses whether these conditions alter *S. cerevisiae* lifespan at least partly by changing rates of genomic instability in young cells. Multiple conditions that increase or decrease lifespan were tested for decreases or increases, respectively, in rates of genomic instability using multiple assays in two strain backgrounds. Overall, there was limited correspondence between these growth conditions and rates of genomic instability in young cells, with the exception of mutations at one locus that were found to predominantly result from non-allelic recombination events. These recombination events were stimulated much more by DNA replication stress than a general DNA damaging agent. This observation supports other work in yeast showing that DNA replication stress and genomic instability due to recombination may be particularly relevant to aging [37, 49, 50].

## Methods

### Yeast strains and media

Yeast strains were grown using standard rich (YPD) or synthetic complete (SC) medium (each containing 2 % glucose), as well as yeast extract-peptone (YP) medium containing high (10 %) or low (0.2 %) concentrations of glucose or YP medium containing non-fermentable carbon sources (3 % potassium acetate, 2 % ethanol, or 3 % glycerol) [51]. Strains harboring mutant alleles were generated by one-step gene replacement or disruption using lithium acetate transformation. Strain SCE218 was derived from JC297 in the GRF167 background (*MATα his3∆200 trp1::hisG ura3-167 Ty1his3AI*) [52] by replacing the *trp1::hisG* allele using a *TRP1* PCR product, and then by disrupting the *LEU2* gene using the pRS406 *URA3*-integrating vector [53] harboring a fragment of the *LEU2* open reading frame (positions 153-898) to generate a *leu2-URA3-leu2* allele. These transformants were selected for Trp^+^ or Ura^+^ Leu^-^ phenotypes, respectively. JC5516 is a derivative of BY4741 (*MATa his3∆1 leu2∆0 met15∆0 ura3∆0*) that harbors a partial deletion allele of *kanMX* in place of the *TRP1* open reading frame (*trp1::kanMX∆NsiI*), partial deletion of *CAN1* at its normal location (*can1∆1*, lacking positions 84-1427 of the open reading frame), insertion of *TRP1* on the left arm of chromosome VIII, and insertions of *CAN1*, *HIS3*, and *URA3* on the right arm of chromosome VIII, as described previously [35]. Derivatives of JC5516 deficient for the *RAD52* gene were generated by replacing the wild type allele with a *kanMX* allele amplified from the *S. cerevisiae MATα* deletion collection (Thermo Scientific Open Biosystems). PCR was used to verify presence of the deletion allele and absence of the wild type allele, and three independent transformants were analyzed.

### Genomic instability rates

Fluctuation tests [54] to measure rates of mutations, recombination, or retrotransposition per cell generation were conducted by inoculating 5,000 cells into each of seven to ten one ml samples of each type of liquid medium indicated in the figures and growing cells in culture tubes on rotators for three days at 20 °C or for 24 h at 30 °C. Cells inoculated into media containing 200 μM (0.0022 %) methyl methanesulfonate (MMS) or 30 mM hydroxyurea (HU) were allowed to grow for four days at 20 °C or 28-30 h at 30 °C. Incubations were up to twice as long for 0.2 % glucose medium and medium containing non-fermentable carbons sources (acetate, ethanol, or glycerol) to achieve comparable cell densities. After growth, appropriate dilutions of cells were spread onto solid SC + 2 % glucose medium to determine colony-forming units/ml. Appropriate volumes of each culture were spread onto solid SC + 2 % glucose lacking arginine with 60 μg/ml canavanine to select for mutations in the *CAN1* gene [55], onto SC + 2 % glucose with 1 mg/ml 5-fluoroorotic acid (FOA) to select for mutations in the *URA3* gene [56], onto SC + 2 % glucose lacking leucine to select for recombination at the *leu2-URA3-leu2* locus to regenerate a *LEU2* allele (see SCE218 strain description), and onto SC + 2 % glucose lacking histidine to select for retrotransposition of a chromosomal Ty1 element harboring the *his3AI* retromobility indicator gene [57], as appropriate for the strain background. Plates were incubated for four days at 30 °C prior to counting colonies. Rate values were determined using the Ma-Sandri-Sarkar maximum likelihood estimator calculated through FALCOR [58], and were corrected based on the fraction of each culture assayed, as previously described [54, 58]. The number of independent trials analyzed for each condition is given in the relevant figure legends.

### Chronological aging experiments

Duplicate cultures of SCE218 or JC5516 were initiated at 5,000 cells/ml in five ml of YPD medium or the alternative media indicated in the figures in glass culture tubes and incubated on a rotator at 20 °C, except for samples grown at 30 °C to test the effects of temperature. Populations were initially sampled every three to five days, and then every seven days until the viability of the control cultures dropped below 10 %. Cell viability was determined by trypan blue dye exclusion, as previously described [35], and three trials were performed for each strain and growth condition.

### Analysis of *CAN1* and *URA3* mutations

Mutations in the *CAN1* and *URA3* genes were categorized as small or large-scale mutations based on PCR or phenotypic screens. Representative colonies from cultures in individual mutation rates trials were patched onto SC-arginine + 2 % glucose + 60 μg/ml canavanine or SC + 2 % glucose + 1 mg/ml FOA, as appropriate. Each derivative tested for strain SCE218 was from an independent culture, and no more than two (*URA3* mutations) or three (*CAN1* mutations) derivatives from given independent cultures were tested for JC5516. PCR for the *CAN1* gene in strain SCE218 or for the *URA3* gene in JC5516 was performed using cells directly as templates or using DNA extracted by glass bead disruption and phenol-chloroform extraction and the following primers (sequences written 5′ to 3′): *CAN1* – GCAAAGGCCACAGAACCGTATTC and GCGAAATGGCGTGGAAATGTGATC, *URA3* – AGCACCCGAACCGAGTGTCCA (matches chromosome VIII sequence nearby *URA3* insertion site) and ACGTTCACCCTCTACCTTAGC. Mutants that yielded a PCR product were scored as having small-scale mutations, and those that did not yield a product were scored as having large-scale mutations. A control primer pair to a single copy gene was included in each PCR to verify that negative results were not due technical failure to amplify a template. The *HIS3* gene is present immediately adjacent to *CAN1* in JC5516 [35], and patches of representative canavanine-resistant colonies from individual cultures in this background were replicated to SC-histidine + 2 % glucose medium to determine whether a functional copy of *HIS3* was present. Patches with a His^+^ prototroph phenotype that retained *HIS3* were considered to have small-scale mutations in *CAN1*, while His^-^ patches were considered to have lost both *CAN1* and *HIS3*. PCR primers used to test for the loss of additional sequences surrounding the site of *CAN1* insertion on chromosome VIII and for non-reciprocal translocations involving sequences from chromosome I included the primers for *URA3* listed above, *HIS3* – ATGACAGAGCAGAAAGCCCTAG and CTTTGGTGGAGGGAACATCGTTG (these primers also amplify the *his3∆1* allele in this strain), *FLO5* – ATGACAATTGCACACCACTGC, ACGCCGCTAACAGTAACGGTG, *IMD2* – GCTCAAGTGGGTCAAAGAGAG and GTAAGAATGTAAATTATGAACG, CTTGGTAGGCTCTTGGTAAAG, *YHRCTy1-1* – GTTCATCATCAGTGATCTGACG, for additional sites on chromosome VIII – AAAGGTTTGTCACGAGCACCG, TGTTGGAATAAGACTCAACTGCGA, CAGCTTCAATGTGGTAAACGGA, GGATTCTCATAAGAAAGTCCAGAG, and for sites on chromosome I – GCCAACATCAATTAATCAACTTTCCC, CGTCTACAGAGTATAGTGTAG, TCGTAGCGAGGACTGCTAAGG, GGATTCTCATAAGAAAGTCCAGAT.

### Statistical analyses

Significant differences in mean rates compared to control values were determined using unpaired two-tailed t-tests assuming equal or unequal variance, as appropriate for the data being compared. Fisher’s exact test was used to determine if ratios were significantly different. Levels of significance are indicated in the figure legends.

## Results

### Not all lifespan-altering growth conditions produced expected changes in chronological lifespan of two strains from different backgrounds aged at 20 °C

Many growth conditions previously reported to alter lifespan did not yield expected changes in mutation rates in young cell populations in initial experiments, so the chronological lifespans of the SCE218 and JC5516 strains in all alternative growth conditions were compared to lifespan in control rich (YPD) medium. YPD was chosen as the control medium rather than synthetic medium (SC) that is often used for chronological aging because YPD has a standard glucose concentration (2 %) to avoid calorie restriction or excess in the control condition, and since acetic acid accumulation in SC shortens lifespan [45], and the intent was to include at least some growth conditions that influenced lifespan independently of acetic acid. Cells were grown at 20 °C, rather than the standard 30 °C, for aging and mutation rate experiments, except when temperature was tested. This lower temperature was used since retrotransposition of yeast Ty1 elements is associated with aging and occurs more frequently at 20 °C than at 30 °C [35, 38, 39, 59, 60]. Ty1 retrotransposition is a source of mutations, so use of the lower growth temperature allowed Ty1 to potentially contribute to any changes in mutation rates. The goal was to identify growth conditions that changed the lifespan of these yeast strains relative to growth in YPD at 20 °C and to measure rates of genomic instability using those conditions, rather than to try to recapitulate specific effects observed using growth at 30 °C or in SC medium as a control condition. Prior work has shown that CLS is reduced by a few changes to standard growth conditions, including the presence of a high glucose concentration in either SC or YPD medium [44, 46], growth in SC leading to accumulation of acetic acid [45], and increased growth temperature using SC medium [44]. Replicative and chronological lifespan are both extended by YPD medium with a low glucose concentration [47, 61], CLS is extended in SC medium with a low glucose concentration [44, 45], and CLS is extended in SC medium containing only a non-fermentable carbon source (such as ethanol or glycerol) [44, 45] or SC medium with high osmolarity (such as 0.3 M sodium chloride) [44, 62]. Cell viability was directly determined using trypan blue dye exclusion, which has previously been used to evaluate changes in chronological lifespan and yields relative changes in lifespan similar to those obtained by measuring colony-forming units [35, 63–65].

**Fig. 1:**
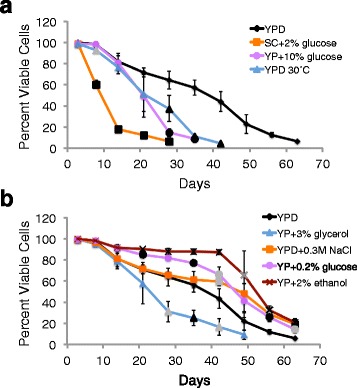
Most growth conditions altered lifespan in strain SCE218 (GRF167 background) as expected. Day zero is when cultures were inoculated, and viability was determined beginning at day three by trypan blue dye exclusion during chronological aging at 20 °C in control YPD medium or in the alternative growth conditions indicated for strain SCE218. **a** and **b** show data for treatments that were expected to shorten or lengthen lifespan, respectively, compared to the same set of data for control medium. Mean and standard deviation of three trials for each condition are shown. Gray or black symbols on the lines for alternative growth conditions indicate time points at which viability differed significantly from the control, with *p* < 0.05 or 0.01, respectively

Most growth conditions resulted in the expected lifespan changes in the SCE218 strain background. Three growth conditions expected to shorten lifespan did result in significantly shorter lifespans in SCE218 (Fig. 1a and Additional file 1: Table S1). Growth in SC + 2 % glucose medium resulted in the shortest lifespan, with a significant drop in viability at all time points beginning with day eight. Growth at 30 °C led to a significant decrease in viability at day eight, and growth at 30 °C and in YP + 10 % glucose both showed significant reductions in viability from day 28 until those populations dropped below 10 % viability. Three of four growth conditions expected to lengthen lifespan resulted in improved viability during chronological aging of strain SCE218 (Fig. 1b and Additional file 1: Table S1). Significantly higher viability was observed at day 21 and most or all time points thereafter for cells grown in YP + 0.2 % glucose or YP + 2 % ethanol, while significantly higher viability was observed at multiple time points beginning with day 42 for cells grown in YPD + 0.3 M NaCl. Growth in YP + 3 % glycerol as a carbon source provided the only exception, which resulted in a significant drop in viability beginning with day 28.

**Fig. 2:**
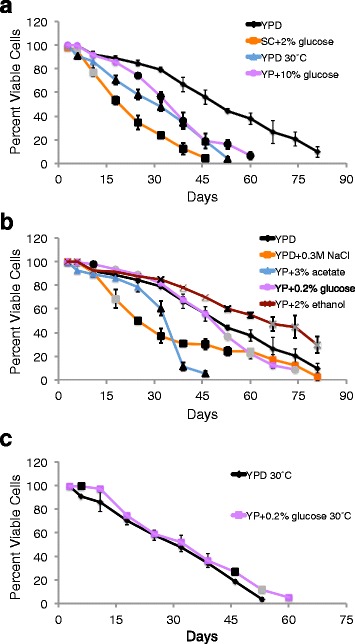
Most alternative growth conditions shortened lifespan in strain JC5516 (BY4741 strain background). Day zero is when cultures were inoculated, and viability for strain JC5516 was determined beginning on day three by trypan blue dye exclusion during chronological aging at 20 °C in control YPD medium or in alternative growth conditions initially expected to shorten **a** or lengthen **b** lifespan is shown. Panel **c** compares the YPD 30 °C data shown in **a** to cells grown in low glucose at 30 °C. Mean and standard deviation of three trials for each condition are shown, and significant differences are indicated as for Fig. 1

The three growth conditions predicted to shorten lifespan had the expected effect on aging of strain JC5516, but fewer treatments extended lifespan of this strain (Fig. 2 and Additional file 1: Table S1). Viability was significantly decreased for JC5516 grown in SC + 2 % glucose, at 30 °C, or in YP + 10 % glucose beginning at day six, six and 18, or 25, respectively, and continuing for the remainder of the experiments (Fig. 2a). However, significantly reduced viability was also observed for cells grown in YPD + 0.3 M NaCl from days 11 to 60 and for cells grown in YP + 3 % potassium acetate beginning at day 32, even though these treatments were expected to lengthen lifespan (Fig. 2b). While growth to stationary phase in acetate medium (as opposed to shifting growing cells or stationary phase cells to medium with acetate) has not previously been reported to extend CLS, acetate was used as an alternative non-fermentable carbon source with JC5516 because of the negative influence of glycerol on the lifespan of strain SCE218. Growth of JC5516 in YP + 0.2 % glucose significantly improved viability at days 11 to 25, but viability then significantly decreased beginning at day 53 (Fig. 2b). Only growth in YP + 2 % ethanol consistently extended lifespan, with significantly increased viability at day 32 and then day 53 through the rest of the time points (Fig. 2b). For the subsequent comparisons to mutation rates, low glucose was categorized as having a positive influence on lifespan because of improved viability early during aging, but any potential influence of growth in low glucose on mutation rates would be expected to be much less than the influence of growth in ethanol. Differences from the anticipated outcomes for some alternative growth conditions may be the result of both strain and temperature-specific effects, since growth of JC5516 in YP + 0.2 % glucose at 30 °C modestly but consistently extended lifespan compared to YPD at 30 °C (Fig. 2c).

### No correlation between direct-repeat recombination, retrotransposition, or mutation rates in young cells and growth conditions that alter lifespan in strain SCE218 (GRF167 background)

**Fig. 3:**
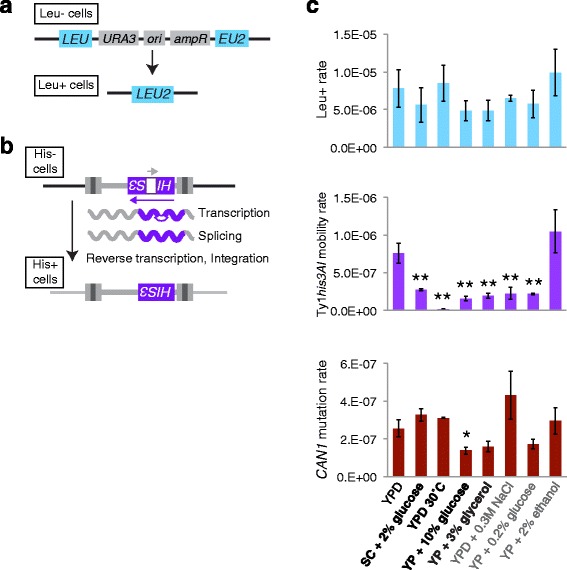
No correlation between recombination, retrotransposition, or *CAN1* mutations and lifespan-altering treatments in strain SCE218. **a** Assay for recombination between two truncated alleles of the *LEU2* gene with overlapping sequences that removes intervening plasmid sequences and restores a wild type *LEU2* allele, conferring a Leu^+^ prototroph phenotype. **b** Assay for retromobility of a chromosomal Ty1*his3AI* element. Expression of Ty1*his3AI* allows splicing of an artificial intron present in the opposite transcriptional orientation of the *HIS3* gene, reverse transcription, and insertion of a Ty1*HIS3* cDNA into the genome, conferring a His^+^ prototroph phenotype. (**c**) Rate values for the indicated forms of genomic instability obtained from fluctuation tests for cells grown for three days in control YPD broth at 20 °C or using the indicated conditions that shortened (bold) or lengthened (gray) lifespan in the SCE218 strain. Mean and standard deviation for four control trials and two trials in each alternative condition are shown. Asterisks indicate level of significance: * = *p* < 0.05, ** = *p* < 0.01

Strain SCE218 harbors a wild type allele of the counter-selectable *CAN1* gene at its normal location on the left arm of chromosome V, truncated and partially duplicated alleles of *LEU2* interrupted by *URA3* and plasmid sequences at the normal location of *LEU2* on the left arm of chromosome III (Fig. 3a), and a chromosomal copy of a Ty1 retrotransposon marked with the *his3AI* retromobility indicator gene [57] (Fig. 3b). This strain can be used to quantify loss-of-function mutations in *CAN1* by detection of canavanine-resistant cells and direct-repeat recombination by detection of Leu^+^ prototrophs resulting from recombination between the truncated *leu2* alleles to reconstitute a wild type *LEU2* allele (Fig. 3a). Mobility of the Ty1*his3AI* retrotransposon can be detected when the intron within *his3AI* is spliced out during Ty1 replication and a reverse-transcribed Ty1*HIS3* element inserts into the genome, giving rise to a cell that is a His^+^ prototroph (Fig. 3b).

Recombination rates to reconstitute *LEU2* were not significantly altered by any of the growth conditions tested, a number of conditions significantly reduced Ty1*his3AI* mobility, and only one condition significantly altered *CAN1* mutation rate (Fig. 3c and Additional file 2: Table S2). All four conditions that shortened SCE218 lifespan reduced the rate of Ty1*his3AI* retromobility, but two of the three conditions that lengthened SCE218 lifespan also reduced Ty1 mobility (Fig. 3c). The similarity in the effects of both types of conditions does not support a role for changes in Ty1 retromobility mediating the lifespan effects of these treatments. Analysis of 28 independent canavanine-resistant derivatives from cells grown in YPD and 19-20 independent derivatives for cells grown in each of the other growth conditions for the presence of the *CAN1* gene resulted in amplification of a *CAN1* product of the expected size from 89-100 % of derivatives (Additional file 3: Table S3), indicating that small-scale mutations were present in nearly all cases. Overall, growth conditions that alter lifespan did not produce expected changes in mutation rates in young cells for strain SCE218.

### Mutation rate at one locus in strain JC5516 (BY4741 background) shows a strong correlation with growth conditions that alter the rate of aging

**Fig. 4:**
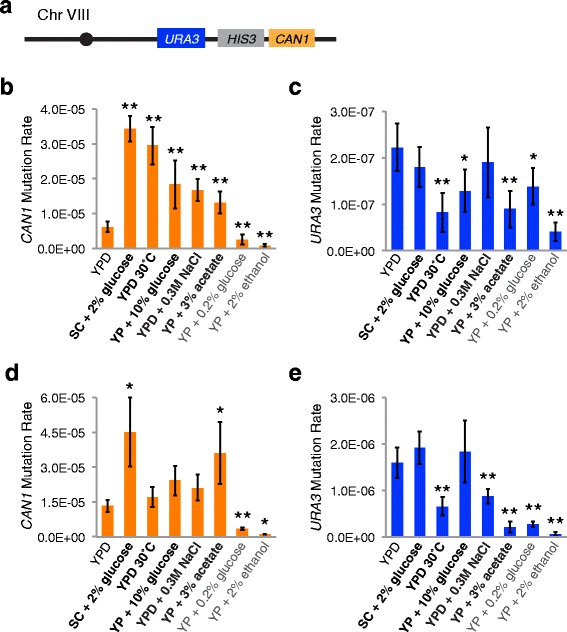
*CAN1* mutation rates in strain JC5516 are strongly correlated with lifespan-altering treatments. **a** Illustration of the relative positions of *URA3*, *HIS3,* and *CAN1* genes inserted onto the distal portion of the right arm of chromosome VIII in the JC5516 strain. **b** *CAN1* mutation rates after growth for three days in control YPD broth at 20 °C or in the indicated growth conditions that were found to shorten lifespan (bold) or lengthen lifespan (gray) of strain JC5516. **c** *URA3* mutation rates after growth for three days in control YPD broth at 20 °C or the same conditions as for panel **b. d** and **e** *CAN1* **d** or *URA3* **e** mutation rates after four days of growth in the same conditions as panels **a** and **b** with chronic exposure to 200 μM (0.0022 %) MMS. Mean and standard deviations for six or seven trials in YPD and three to five trials in alternative media conditions (**b** and **c**) or three to four trials with MMS treatment (**d** and **e**) are shown. Note the change in y-axis scales between panels **b** and **d** and panels **c** and **e**. Asterisks indicate level of significance: * = *p* < 0.05, ** = *p* < 0.01

Rates of *CAN1* and *URA3* mutations in growth conditions that alter lifespan were measured using strain JC5516. This strain has a complete deletion of *URA3* and a partial deletion of *CAN1* from their normal genomic locations, and insertions of *URA3* and *CAN1* approximately 40 kbp and 26 kbp from the end of the right arm of chromosome VIII, respectively [35] (positions 522,206 and 536,834 on chromosome VIII in the reference *S. cerevisiae* genome [66], Fig. 4a).

*CAN1* mutation rates were significantly different in all lifespan-altering growth conditions tested (Fig. 4b and Additional file 2: Table S2). The five conditions that were shown to reduce JC5516 viability during chronological aging all increased the *CAN1* mutation rate from 2.1 to 5.5-fold. The largest increase was observed for SC + 2 % glucose, which had the strongest negative effect on lifespan, followed by YPD at 30 °C, which also quickly led to decreased viability in the lifespan experiments (Fig. 4b and Fig. 2a). The smaller effects of YP + 10 % glucose, YP + 3 % acetate, or YPD + 0.3 M NaCl on *CAN1* mutation rate are consistent with their less negative influences on lifespan. Growth in YP + 2 % ethanol reduced the *CAN1* mutation rate 7.7-fold, consistent with the positive influence of ethanol on lifespan, while growth in YP + 0.2 % glucose only reduced the rate 2.5-fold, consistent with the very modest positive influence of low glucose early during lifespan in JC5516 (Fig. 4b and Fig. 2a).

The *CAN1* mutation rate was much higher (24-fold) when *CAN1* was located on chromosome VIII in JC5516 than when it was at its normal location on chromosome V in SCE218 (compare Fig. 3c and Fig. 4b). Only 24 % of JC5516 canavanine-resistant derivatives from control cultures retained function of the neighboring *HIS3* gene, which was also inserted onto the right arm of chromosome VIII in this strain [35] (Fig. 4a), as determined by transferring cells to medium lacking histidine (Table 1). This indicates that most of these mutations were not small-scale changes in the *CAN1* locus. The frequency of small-scale *CAN1* mutations was modestly elevated for cells grown in YP + 2 % ethanol, but was unchanged in the other growth conditions (Table 1).

**Table 1:** Mutations in *CAN1* are mostly large-scale changes, while mutations in *URA3* are mostly small-scale changes

Sample	*CAN1* mutations				*URA3* mutations			
	Untreated		+ MMS^*a*^		Untreated		+ MMS^*a*^	
	*N*	Freq His^+^	*N*	Freq His^+^	*N*	Freq point Mutations^*b*^	*N*	Freq point mutations^*b*^
YPD	217	0.24	110	0.46	99	0.95	43	0.98
SC + 2 % Glucose	137	0.24	80	0.41	57	1.0	13	0.92
30 °C	102	0.28	66	0.47	27	0.96	16	1.0
10 % Glucose	111	0.21	83	0.46	33	0.97	50	0.94
0.3 M NaCl	135	0.26	81	0.41	46	1.0	39	0.97
3 % Acetate	98	0.18	78	0.33	37	0.81*	19	1.0
0.2 % Glucose	106	0.27	82	0.38	38	0.87	50	0.96
2 % Ethanol	108	0.39**	96	0.34	46	0.80*	40	0.95
YPD *rad52∆*	59	0.88***	NT^*c*^	NT^*c*^	25	0.96	NT^*c*^	NT^*c*^

^*a*^Chronic exposure to 200 μM (0.0022 %) MMS

^*b*^Scored as point mutation if yielded expected size *URA3* PCR product

^*c*^Not tested

Asterisks indicate levels of significance: * = *p* < 0.05, ** = *p* < 0.01, *** = *p* < 0.001

*URA3* mutation rates were significantly reduced by both conditions that had a positive influence on lifespan in JC5516, but were also reduced by three of the conditions that shortened lifespan (Fig. 4c and Additional file 2: Table S2). None of the conditions that shortened lifespan increased the *URA3* mutation rate. Decreases in the rate ranged from 1.7 to 5.5-fold, with growth in YP + 2 % ethanol producing the greatest decrease. PCR for the presence of *URA3* with cells or DNA extracted from representative FOA-resistant derivatives from each of the growth conditions indicated that nearly all mutations in *URA3* were small-scale (Table 1). This is similar to what was observed for *CAN1* in SCE218, but contrasts with the results for *CAN1* in JC5516. Modest but significant decreases in the proportion of *URA3* mutants with small-scale changes were observed when cells were grown in YP + 2 % ethanol or YP + 3 % acetate (Table 1).

**Fig. 5:**
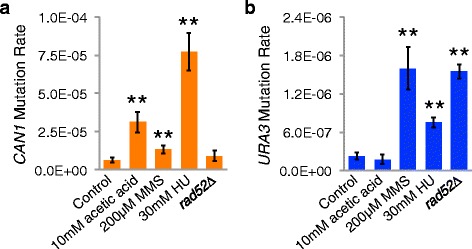
*CAN1* mutations in JC5516 are greatly elevated by DNA replication stress. Mean and standard deviation for *CAN1* (**a**) or *URA3* (**b**) mutation rates in JC5516 grown for three days at 20 °C (or four days for HU and MMS) in standard rich YPD medium (Control), in YPD with chronic exposure to 10 mM acetic acid, 30 mM HU, or 200 μM MMS, or in three *rad52∆* derivatives grown in YPD. Control and MMS data are the same as shown in Fig. 4. Results of three to four trials are shown for treatments and *rad52∆* mutants. Double asterisks indicate *p* < 0.01 versus control

*CAN1* and *URA3* mutation rates were then compared following chronic exposure to 200 μM (0.0022 %) MMS in all growth conditions to determine if the alternative growth conditions would exacerbate or ameliorate the effects of an exogenous DNA damaging agent (Fig. 4d and e, Additional file 4: Table S4). This concentration of MMS caused only a modest decrease in growth rate of the cells, requiring four days of growth at 20 °C in YPD to achieve similar cell densities to those obtained after three days of growth without MMS. Treatment with MMS caused a significant 2.1-fold increase in *CAN1* mutation rate in YPD medium (compare Fig. 4b and d and see Fig. 5a). Only SC + 2 % glucose and YP + 3 % acetate led to further significant increases in the *CAN1* mutation rate, of 3.4 and 2.7-fold, respectively (Fig. 4d). The other three conditions determined to shorten lifespan in JC5516 led to further increases of less than two-fold that were not significant. Growth in YP + 0.2 % glucose or YP + 2 % ethanol decreased *CAN1* mutation rate in the presence of MMS by 3.9 or 12-fold respectively, which were greater fold-decreases than observed for these growth conditions in the absence of MMS (compare Fig. 4b and d). Overall, four of the seven conditions exacerbated or ameliorated the effect of MMS. The influences of the growth conditions that shortened lifespan were less consistent, though, than what was observed in the absence of MMS treatment.

MMS treatment caused a 7.2-fold increase in the *URA3* mutation rate, which is substantially greater than the increase observed with the *CAN1* mutation rate (Fig. 5a and b). None of the conditions shown to shorten JC5516 lifespan further increased the MMS-induced *URA3* mutation rate, and growth at 30 °C, in YPD + 0.3 M NaCl, or in YP + 3 % acetate instead caused 2.4, 1.9, or 7.5-fold decreases in the MMS-induced *URA3* mutation rate, respectively (Fig. 4e). Growth in YP + 0.2 % glucose or YP + 2 % ethanol did lead to greater reductions in the MMS-induced *URA3* mutation rate, of 5.9 and 24-fold, respectively, than the corresponding reductions observed without MMS-treatment (Fig. 4c and e). Exposure to MMS did not lead to an improved correspondence between the growth conditions and their predicted effects on *URA3* mutation rate, considering that the lifespan-shortening conditions did not lead to increases in the mutation rate in the absence or presence of MMS.

### *CAN1* mutations in JC5516 are large-scale recombination events

The types of mutation events leading to *CAN1* mutations in JC5516 were explored in more detail, since the mutations occurred at a high rate and growth in alternative conditions yielded expected changes in the *CAN1* mutation rate. The preliminary analysis shown in Table 1 indicated that most of the *CAN1* mutations were large-scale events that resulted in loss of function of *CAN1* and the neighboring *HIS3* gene. Deletions of chromosome segments are usually rare compared to small-scale mutations, but homologous recombination-based events could potentially delete/replace the *CAN1* and *HIS3* sequences at a relatively high rate. Since JC5516 is a haploid strain, homologous recombination events would be expected to require the presence of suitable non-allelic homologous sequences to delete or replace the relevant region of chromosome VIII. Recombination-based events might also be more sensitive to DNA replication stress, since template switching to non-allelic homologous sequences during replication could give rise to such events [67].

Growth in SC + 2 % glucose leads to shortened lifespan due to accumulation of acetic acid in the medium, and suspension of stationary phase yeast cells in water containing 10 mM acetic acid shortens their lifespan relative to suspension in water only [45]. Acetic acid has also been shown to increase DNA replication stress in yeast [68]. Growth of JC5516 in YPD with 10 mM acetic acid increased *CAN1* mutation rate five-fold, but did not increase the *URA3* mutation rate (Fig. 5a and b), similar to the results for growth in SC + 2 % glucose (Fig. 4b and c). Cells were also exposed to 30 mM hydroxyurea (HU) to induce a moderate DNA replication stress throughout the growth period, which resulted in a 12-fold increase in *CAN1* mutation rate, but only a 3.4-fold increase in *URA3* mutation rate (Fig. 5a and b). These results contrast with those of the DNA alkylating agent MMS, which led to only a 2.1-fold increase in *CAN1* mutation rate, but a 7.2-fold increase in *URA3* mutation rate. The increased mutation events with MMS treatment were also associated with an increased proportion of small-scale mutations in YPD and most alternative growth conditions (Table 1), indicating that MMS treatment had little affect on the events leading to simultaneous loss of *CAN1* and *HIS3* function. These data are consistent with problems during DNA replication contributing to the high rate of *CAN1* mutations in JC5516.

Loss of *RAD52* function compromises homologous recombination and produces a hypermutation phenotype that is characterized by an increase in base substitutions [69, 70]. JC5516 *rad52∆* derivatives had a seven-fold increase in the *URA3* mutation rate but no significant change in the *CAN1* mutation rate (Fig. 5a and b). Furthermore, most of the *CAN1* mutations in the *rad52∆* strains were small-scale changes, based on retention of the function of the neighboring *HIS3* gene, in contrast to the low frequency of small-scale changes in the *RAD52* parent strain (Table 1). The increase in the proportion of small-scale changes and the absence of a change in the *CAN1* mutation rate indicate that loss of *RAD52* decreased the rate of large-scale changes causing loss of *CAN1* function. The combined data from *rad52∆* mutants, acetic acid treatment, and HU treatment are consistent with the *CAN1* mutations in JC5516 largely arising from recombination events associated with DNA replication stress.

**Fig. 6:**
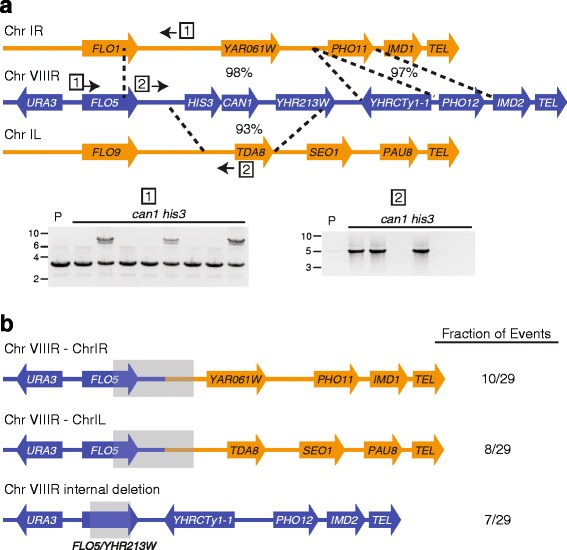
Loss of *CAN1* in JC5516 frequently results from non-reciprocal translocations or interstitial deletions. **a** Drawings of relative positions and orientations of selected gene, pseudogene, and telomeric sequences (*TEL*) flanking the site of insertion of *CAN1* and *HIS3* on the right arm of chromosome VIII (blue), as well as selected features for relevant regions from the right and left arms of chromosome I (orange). Dotted vertical lines with percentages indicate sequence identity between regions of chromosomes VIII and I. Boxed numbers and small arrows indicate the relative positions of representative primers used to test for chromosome VIII-I translocations. Representative images of PCR products from the parent strain (P) and independent *can1his3* derivatives visualized by staining agarose gels with ethidium bromide (signal inverted) are shown for the two different pairs of primers. The translocation product is the upper product in the left image, and the lower product was generated with a control primer pair to the *HSP104* gene at an unrelated chromosome region. Numbers to the left of the images indicate the positions of molecular mass markers in kbp. **b** Drawings of the most common non-allelic recombination events identified and their frequency in the total set of events. Gray boxes at the junctions indicate that the precise transitions between *FLO5* sequences or sequences between *FLO5* and the site of *HIS3* on chromosome VIII and chromosome I sequences varied amongst the independent samples. The blue arrow with the gray box for the third class of events indicates a fusion between *FLO5* and the *YHR213W* pseudogene at various sites within each sequence. Sequence features are from the *S. cerevisiae* reference genome [66]

The presence or absence of nearby sequences on chromosome VIII was examined by PCR with a set of 29 independent *can1* mutants that were unable to grow on medium lacking histidine. As expected based on their His^-^ phenotype, all 29 mutants failed to yield a product corresponding to the *HIS3* allele that was originally present immediately centromere-proximal to *CAN1*. All 29 *can1his3* mutants retained the *URA3* gene approximately 15 kbp centromere-proximal to *CAN1*. During analysis of these events, it was noticed that a nearly 16 kbp region encompassing the site at which *HIS3* and *CAN1* were inserted onto chromosome VIII is a 98 % sequence match to a region near the right arm telomere of chromosome I [66] (Fig. 6a). Approximately 5 kbp more sequence after a Ty1 insertion in this region of chromosome VIII, *YHRCTy1-1*, is a 97 % sequence match to the region of chromosome I, which lacks a Ty1 element (Fig. 6a). In addition, an approximately four kbp region near the left arm telomere of chromosome I is a 93 % match to the region of chromosome VIII encompassing the *CAN1* and *HIS3* insertions [66] (Fig. 6a). Primers capable of distinguishing between the relevant chromosome VIII and I regions were used to determine whether non-allelic recombination between these two chromosomes contributed to the *CAN1* mutations (example PCR results are shown in Fig. 6a).

This PCR analysis identified ten (34 % of events) or eight (28 % of events) non-reciprocal translocations between chromosome VIII and either the right arm or left arm region of chromosome I, respectively (Fig. 6b and Additional file 5: Table S5). These events resulted in fusion of chromosome VIII and I sequences, as well as the loss of the terminal region of the right arm of chromosome VIII, but the translocations occurred at multiple regions of chromosome VIII in each category (Fig. 6b and Additional file 5: Table S5). Precise sites for the transition from chromosome VIII to chromosome I sequences could not be determined because of long stretches of identical sequences on the two chromosomes around the site of the insertion of *CAN1* and *HIS3* [66]. Two additional events involved non-reciprocal recombination with the chromosome I regions, but distal sequences from chromosome VIII were retained (*YHRCTy1-1* and *IMD2*), consistent with gene conversion recombination with the chromosome I regions (Additional file 5: Table S5). Another two events were not missing any chromosome VIII sequences other than *CAN1* and *HIS3*, but also likely resulted from gene conversion recombination with chromosome I at sites within the sequences that are completely identical between the two chromosomes. Including these last two cases, 22 of the 29 events (76 %) resulted from non-allelic recombination with chromosome I (Additional file 5: Table S5).

The remaining seven events (24 %) had internal deletions on the right arm of chromosome VIII due to recombination between the *FLO5* gene and the *YHR213W* pseudogene that has substantial sequence similarity to *FLO5* [66] (Fig. 6b and Additional file 5: Table S5). Junctions occurred between chromosome VIII positions 525,819-526,286 in *FLO5* and positions 539162-539629 in *YHR213W* in the reference genome [66]. All 29 of the events can therefore be explained by non-allelic recombination, indicating that the high frequency of *CAN1* mutations in the JC5516 strain is primarily the result of homologous recombination.

## Discussion

The results of only one of five genomic instability assays using two different strain backgrounds was consistent with lifespan-altering growth conditions changing the rate of aging by altering mutation rates in young cells. These results do not contradict data from numerous studies showing that DNA damage and mutations increase with age, since these prior studies in general did not test whether increased DNA damage and mutation frequencies were directly responsible for aging [6, 71]. The results are also consistent with a recent report that mutation accumulation does not appear to be responsible for limiting the replicative lifespan of dividing yeast mother cells, based on analysis of daughter cell lineages from mothers of different ages [72]. The current study focused on mutations producing a phenotypic effect, rather than all mutations that could be detected by DNA sequencing, since it is likely that mutations would need to have a phenotypic effect at some level in order to affect lifespan.

Not all longevity-altering growth conditions produced the expected effects on lifespan, particularly for strain JC5516. This may be due to strain-specific differences, but also could result from using growth in YPD at 20 °C as the control condition. Many alternative growth conditions that alter chronological aging have been tested in comparison to growth in SC at 30 °C [44, 45], and medium acidification is a major determinant of lifespan in SC [45], but not in YPD [46]. For instance, the beneficial effect of glycerol on lifespan in SC is at least partly due to reduced medium acidification but ethanol exposure did not reduce acidification [45], which could explain why glycerol did not extend lifespan similarly to ethanol in rich medium. The increase in yeast CLS observed with growth at temperatures below 30 °C could also have masked or changed the influence of some alternative growth conditions [35, 44]. In addition, acetate was expected to increase CLS because it is a non-fermentable carbon source, but addition of acetate to stationary phase yeast cells in water has been reported to shorten lifespan [73]. Despite some unanticipated lifespan results, it was still possible to test conditions that increased or decreased CLS for influences on mutation rates.

The replication of yeast Ty1 retrotransposons has been associated with elevated genomic instability during chronological aging [35], and retrotransposons from multiple organisms are more highly expressed or mobile with increased age [74–78]. However, no correlation was observed between lifespan-altering conditions and Ty1 mobility in the current data, and it was recently reported that strains with Ty1 retrotransposons did not have shorter CLS than a strain lacking Ty1 retrotransposons when grown in standard rich medium [65]. Ty1 retromobility is also correlated with genomic instability during replicative aging of mother cells, but a direct role for Ty1 in shortening replicative lifespan has not yet been tested [38].

The *CAN1* mutation rate in JC5516 was strongly correlated with the lifespan effects of the growth conditions. *CAN1* mutation events in JC5516 arose from recombination between non-allelic genomic sites. Many such events could have resulted from use of break-induced replication (BIR) for double-strand break repair or restart of a stalled replication fork, resulting in a DNA replication fork leaving the chromosome VIII template and resuming DNA synthesis on a homologous region of chromosome I or VIII to produce gene conversion and non-reciprocal translocation events or internal deletions, respectively [67]. Alternatively, the interstitial deletions of chromosome VIII that were observed could have resulted from single-stranded annealing (SSA) between homologous sequences flanking *CAN1* during DNA repair or replication [67, 79].

The results for the chromosome VIII recombination events in JC5516 contrast with the absence of a correlation between direct-repeat recombination events and the alternative growth conditions in strain SCE218. One difference between these recombination events is that identical sequences were involved in the *LEU2* recombination events, while the sequences involved in the *CAN1* mutation events were only 93-98 % homologous. The Sgs1p protein in yeast inhibits recombination between sequences that are not identical [80, 81]. Mutants with a deletion of *SGS1* exhibit elevated genome instability during chronological aging, reduced CLS with extreme calorie restriction, and reduced replicative aging [48, 82, 83]. Differential expression or activity of Sgs1p, or other DNA repair factors that suppress recombination between non-identical sequences, in response to the alternative growth conditions could account for the different results for the two types of recombination events.

Prior work has shown that loss of heterozygosity due to recombination events and large-scale chromosomal alterations increases during yeast replicative aging [36, 37, 39], and that increased DNA replication stress is correlated with decreased yeast CLS [49, 50]. Several studies have noted that large-scale chromosomal abnormalities, including loss of heterozygosity through interstitial deletions or conversion of one chromosome region by the corresponding region from the homologous chromosome, increase with age in humans [22–25]. In particular, one group noted that their results were consistent with roles for BIR and SSA [22]. The correlation between chromosome changes that likely arise through BIR and SSA and lifespan-altering treatments in yeast indicates that future work in yeast has the potential to provide insights into mechanisms and regulation of chromosomal abnormalities that may prove relevant to human aging.

## Conclusions

These data indicate that changes in the overall accumulation of various mutations early during lifespan are not likely to be directly responsible for the influences of the growth conditions tested on yeast lifespan, but do support the role of DNA replication stress and recombination-based genome rearrangements in yeast aging. It is unclear if the occurrence of homologous recombination-dependent chromosomal deletions and translocations is a direct determinant of lifespan or simply serves as a sign of increased DNA replication stress. Increased recombination is observed in aging yeast mother cells [37] and DNA replication stress is associated with decreased yeast chronological lifespan [49, 50]. DNA replication stress reduces hematopoietic stem cell function in aging mice [84], and mutations that underlie multiple human segmental progeroid disorders lead to defective DNA replication and chromosomal instability [85–87]. Furthermore, oncogene induced replication stress occurs during early stages of cancer progression and promotes chromosomal deletions and translocations through non-allelic recombination [88]. The current results emphasize the importance of pursuing the role of DNA replication and recombination-based chromosomal instability in aging.

## Additional files

Additional file 1: Table S1. Results of individual chronological lifespan trials for two strain backgrounds in media conditions expected to alter lifespan. (XLSX 43 kb)
Additional file 2: Table S2. Mutation rates for individual trials in two strain backgrounds in media conditions that alter lifespan. (XLSX 44 kb)
Additional file 3: Table S3. Fraction of canavanine-resistant derivatives in SCE218 that yielded a *CAN1* PCR product. (XLSX 27 kb)
Additional file 4: Table S4. Mutation rates for individual trials of JC5516 treated with MMS, acetic acid, HU, or in *rad52∆* mutants. (XLSX 44 kb)
Additional file 5: Table S5. Characterization of 29 large-scale *CAN1* mutation events in JC5516. (XLSX 44 kb)

## Abbreviations

BIR: Break-induced replication
CLS: Chronological lifespan
HU: Hydroxyurea
kbp: Kilobase pair
MMS: Methyl methanesulfonate
SSA: Single-stranded annealing
